# Irisin/FNDC5 Regulates Endothelial Function to Improve Post‐Stroke‐Induced Cognitive Dysfunction by Stimulating AMPK‐eNOS Signaling

**DOI:** 10.1002/brb3.70767

**Published:** 2025-09-15

**Authors:** Hui‐Hui Guo, Jun‐Jie Liang, Rui‐Huan Pan, Mei‐Feng Zheng, Ya‐Xian Qiu, Shan‐Shan Jiang, Xin‐Yu Fu, Hector Wing‐Hong Tsang, Suk‐Yu Yau, Hai‐Ning Ou

**Affiliations:** ^1^ Department of Rehabilitation Medicine Shaoxing People's Hospital Shaoxing China; ^2^ Department of Rehabilitation Medicine, Panyu Central Hospital Guangzhou Medical University Guangzhou China; ^3^ Department of Rehabilitation The Second Affiliated Hospital of Guangzhou University of Chinese Medicine Guangdong Provincial Hospital of Chinese Medicine Guangzhou China; ^4^ The Second Institute of Clinical Medicine Guangzhou University of Chinese Medicine Guangzhou China; ^5^ Key Laboratory of Biological Targeting Diagnosis, Therapy and Rehabilitation of Guangdong Higher Education Institutes, The Fifth Affiliated Hospital Guangzhou Medical University China; ^6^ Department of Rehabilitation Sciences The Hong Kong Polytechnic University Kowloon Hong Kong China; ^7^ Mental Health Research Center The Hong Kong Polytechnic University Hong Kong China

**Keywords:** cognitive impairment, FNDC5, ischemic stroke, irisin, physical exercise

## Abstract

**Background:**

Cognitive impairment is one of the main complications after a stroke and seriously affects the quality of life and survival time of patients, thereby causing a heavy burden on the social economy and public health. Although exercise is an effective non‐pharmacological strategy for prevention and treatment of cognitive impairment, the mechanism(s) of this effect remain unclear.

**Methods:**

The current study investigated the effects of irisin treatment on the behavioral characteristics of mice with post‐stroke cognitive impairment (PSCI). The expression levels of platelet endothelial cell adhesion molecule 1 (PECAM, PECAM‐1, CD31), glial fibrillary acidic protein (GFAP), vascular endothelial growth factor (VEGF), and molecules in the adenosine 5‐monophosphate (AMP)‐activated protein kinase (AMPK)–endothelial nitric oxide synthase (eNOS) signaling cascade in the hippocampus were then measured.

**Results:**

Irisin significantly enhances learning and memory functions in cases of PSCI. This improvement correlates with a reduction in cerebral infarction size and decreased neuronal death. Additionally, irisin treatment resulted in a marked decrease in the levels of astrocytic scar formation in the cortex. Furthermore, irisin activates the AMPK‐eNOS signaling pathway, which promotes the expression of VEGF. The irisin compounds are involved in the process of brain angiogenesis and play a critical role in endothelial and reactive astrocytes function.

**Conclusion:**

The study revealed a potential mechanism by which exercise‐induced irisin secretion may attenuate PSCI. Irisin improved endothelial dysfunction and neuroinflammation, suggesting it may be a promising target for PSCI therapy.

## Graphical abstract

Irisin/FNDC5 is a crucial regulator of the cognitive benefits of exercise in post‐stroke cognitive impairment. The following schematic diagram shows that increasing the irisin/FNDC5 level can activate the AMPK‐eNOS pathway, promoting lower infraction volume and learning and memory cognitive functions.

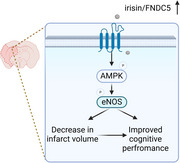

## 1 Introduction

Stroke is the major cause of death and disability in China (Tu et al. 2023), with approximately 10%–30% of patients developing post‐stroke cognitive impairment (PSCI) (Craig et al. 2022). To date, there are no useful drugs for the clinical treatment of this condition. However, studies in humans have indicated that exercise may be a potential method for preventing and treating cognitive impairment in older people (Tarumi et al. 2022; Chen et al. 2023; Azevedo et al. 2023). The benefits of exercise include promoting the release of neurotrophins (Bastioli et al. 2022), increasing the rate of adult hippocampal neurogenesis (Leiter et al. 2022; Yau et al. 2014), attenuating neuroinflammation (Barad et al. 2023; De Miguel et al. 2021), modulating cerebral blood flow (CBF) (Khan et al. 2023; Tomoto et al. 2023), and promoting neuronal plasticity (Hill et al. 2023; Marino et al. 2023). Exercise also promotes social interaction with beneficial cognitive outcomes (Jia et al. 2023).

Irisin is a hormone, which is cleaved from the fibronectin type III domain containing 5 (FNDC5) (Lourenco et al. 2019), produced mainly by muscle cells and adipocytes, but also expressed widely in brain tissue. The hormone has an anti‐neuroinflammation effect in addition to regulating glucose and lipid metabolism, controlling vasodilation, and protecting nerves, changes that improve memory and cognition (Liu et al. 2022; Bao et al. 2022). Clinical studies have reported that serum irisin levels are reduced in patients with the first acute stroke (Tu et al. 2018; Wu et al., 2019), while in Alzheimer's disease (AD) animal models, it has been shown that FNDC5/irisin levels are also decreased in both the hippocampus and cerebrospinal fluid (Lourenco et al. 2019). There is also evidence that exercise training significantly increases the expression level of brain‐derived neurotrophic factor (BDNF) in the hippocampus by activating the peroxisome proliferator‐activated receptor‐γ co‐activator‐1α (PGC‐1α)/FNDC5/BDNF pathway (Wrann et al. 2013). In contrast, knockdown of brain FNDC5/irisin leads to learning and memory impairment, whereas a boost in its levels improves synaptic function and memory capacity (Wrann et al. 2013; Lourenco et al. 2019).

Although many studies have suggested that irisin may have beneficial protective effects on brain injury and cognitive deficits (Islam et al. 2021; Li et al. 2017; Wang et al. 2022), the nature of these effects and their specific molecular mechanisms in a PSCI model are unknown. The present study, therefore, measured the effects of irisin in a mouse model of PSCI and investigated possible underlying mechanism(s) for these effects.

## 2 Materials and Methods

All the studies on the animals were carried out in accordance with the Chinese and the European Communities Council Directive of September 20, 2010 (2010/63/EU) for the Experimental Animals Administration Legislation. The study was approved by the Hong Kong Polytechnic University (PolyU) Shenzhen Research Institute Committee on Animal Care.

### 2.1 Animals

Note that 6‐ to 8‐week‐old adult male C57BL/6J mice were purchased from Guangdong Medical Laboratory Animal Center, China (license No. SCXK [Yue] 2023‐0072). The mice were then transferred to a feeding room with a controlled temperature (23 ± 1°C) and humidity (55 ± 2%) and a 12‐h light/dark cycle (8:00 a.m.–8:00 p.m.). The animals had free access to food and water.

### 2.2 Experimental Protocol

The mice were divided randomly into three groups: (a) sham, (b) middle cerebral artery occlusion (MCAO), and (c) MCAO + irisin (0.2 mg/kg and/or 0.2 µg/g) (Li et al. 2017). The surgical technique to cause an MCAO was performed as described previously (Longa et al. 1989; Guo, Zhu et al. 2021). Irisin was administered by tail vein injection 30 min after MCAO surgery, while the control groups were given the same volume of vehicle (0.9% saline). Twenty‐four hours after the stroke, neurological scores, infarct volume, and mouse body weight were measured, and immunofluorescence staining, Western blotting, and ELISA assays were performed to investigate the relevant molecular mechanisms. Finally, the brains were obtained, and fresh hippocampus tissue and serum were collected and stored at −80°C until further use.

### 2.3 PSCI Model

The MCAO was used to induce the PSCI model in this study. Briefly, prepare 8‐0 nylon thread into 1‐ to 1.5‐cm pieces before the surgery. Administered 5% isoflurane in a mixture of 30% O_2_ and 70% N_2_O using the isoflurane anesthesia system (RWD, Shenzhen, China) for deep anesthesia. Subsequently, the concentration of isoflurane was decreased and stabilized at 1%. A midline neck incision (1–1.5 cm) was made, and the right common internal carotid artery was exposed. The external carotid artery was ligated, and a slipknot was tied at the proximal ends. The internal carotid artery was clamped, and a silicone suture was inserted into the common carotid artery. The external carotid artery was cut, it was aligned with the internal carotid artery, and the slipknot was tightened. The incision was disinfected and covered, embolization was performed, and after 90 min, the ligature was tightened and the incision was closed with 4‐0 nylon suture (Longa et al. 1989; Guo et al. 2021). The mice's body temperature was kept at 37 ± 0.5°C throughout the procedure, ensuring that they remained stable until they fully recovered from the surgery. Neurological scoring, 2,3,5‐triphenyltetrazolium chloride (TTC) staining, and behavioral tests were performed to validate the effectiveness of the brain ischemia model.

### 2.4 Neurological Scoring

A modified Bederson (0–5) score (Meisel et al. 2004) was used as follows: 0, no neurological impairments (*normal*); 1, decreased extension of the forepaw (*mild*); 2, circling (*moderate*); 3, loss of postural reflexes (*severe*); and 4, death (*critical*). Mice with a score between 1 and 3 were used for the behavioral tests and molecular assays.

### 2.5 TTC Staining

The mice brains were sliced into five pieces, followed by incubation at 37°C for 30 min in 2% (wt/vol) TTC in phosphate buffer. The TTC‐stained slices were digitalized, and the infarct area was then calculated using Image Pro Plus 6.0 software (Media Cybernetics, Maryland, USA).

### 2.6 Behavioral Tests

All the behavioral tests in this study were performed as described previously (Zhang et al. 2021). For the open field test (OFT) that evaluated the autonomous, exploratory, and emotional behaviors, the mice were placed in the center of the bottom of a box (50 cm × 50 cm × 50 cm) to freely explore the area for 5 min. The Y‐maze and novel object recognition (NOR) tests were used to evaluate recognition memory. Both of these tests consisted of two phases: learning and testing. In the Y‐maze test, three identical arms (L × W × H: 10 × 6 × 8 cm^3^) were constructed before learning and testing, and acted as the starting, familiar, and novel arms, respectively. During the habituation session, the mice were allowed to freely explore the maze with the novel arm blocked. After a 2‐h intermission, the blocked novel arm was opened, and the behavior of the mice was recorded for 5 min (i.e., time spent and number of inspections in each arm). The percentage (%) alternation was calculated using the following formula: % alternation = (number of alternations/total number of arm entries − 2) × 100 (Kraeuter et al. 2019). In the NOR test, the mice were first allowed to familiarize themselves with the test box for 15 min on Day 1, the same as that used in the OFT box. On Day 2, the mice were placed into the test box containing two similar cubic objects (7 cm × 7 cm × 15 cm) for 10 min. After a 2‐h intermission, one of the new objects was changed. The following spent (s) time exploring the familiar (F) and the novel (N) objects over a 5‐min period was then recorded. The discrimination index (DI) was calculated as (N − F)/(N + F).

### 2.7 Immunofluorescence Staining

Immunofluorescence staining was used to detect the positive expression of CD31 and GFAP in the PSCI model (Guo et al. 2021). Briefly, the brain tissues collected were fixed with 0.01 M PBS and 4% paraformaldehyde and then transferred progressively to 15%, 20%, and 30% sucrose solutions to dehydrate. The tissues were frozen with OCT and stored at −20°C until being cut into 30‐µm‐thick slices containing the target region of the brain. The slices were washed with 0.01 M PBS for 5 min and incubated with blocking buffer (5% normal goat serum, 0.3% Triton X100) for 1 h at room temperature, followed by incubation at 4°C overnight with primary antibodies targeted against CD 31 (Cat No. 3528S; Cell Signaling Technology, Inc.) and GFAP (Cat No. 16825‐1‐AP; Proteintech Inc., Wuhan, China). After washing, the slices were incubated for 2 h in the dark with a secondary antibody (Cat No. ab150077; ab150113; Abcam). The sections were then sealed with an antifade solution containing DAPI (Cat No. P0131; Beyotime), and the immunofluorescence signals were detected immediately using fluorescence microscopy. Positive staining was captured and analyzed using the 3D HISTECH digital pathology slide scanner and slide viewer software (Pannoramic 1000 and Case Viewer 2.4, 3D HISTECH; Hungary) and ImageJ software.

### 2.8 Western Blotting

Protein was isolated using a protease inhibitor cocktail and phosphatase inhibitors in RIPA buffer (Guo et al. 2021). The protein concentrations were measured using the BCA protein assay kit. Equal amounts of protein (35 µg) were separated by 10% or 12% SDS‐PAGE and then transferred to PVDF membranes. After blocking with 5% BSA for 1 h at room temperature, the membranes were incubated at 4°C overnight with antibodies targeted against: p‐AMPKα (where AMPK stands for AMP‐activated protein kinase) (1:1,000; Cat No. 2535S), AMPKα (1:1,000; Cat No. 10929‐2‐AP), irisin/FNDC5 (1:1,000; Cat No. ab131390), AKT (1:1,000; Cat No. 10176‐2‐AP), p‐AKT (1:1,000; Cat No. ab38449), eNOS (1:1,000; Cat No. 27120‐1‐AP), p‐eNOS (1:1,000; Cat No. ab184154), vascular endothelial growth factor VEGF (1:1,000; 26157‐1‐AP), and GAPDH (1:5,000; Cat No. 10494‐1‐AP). Following washing of the membranes with TBST (0.1% Tween‐20) and incubation with the appropriate secondary antibody for 2‐h at room temperature, the protein bands displayed were quantified using an imaging apparatus (Bio‐Rad, Hercules, California, USA).

### 2.9 ELISA

Irisin/FNDC5 concentrations were determined in both serum and hippocampus tissue. The levels of irisin/FNDC5 (irisin/FNDC5 ELISA Kit; cat. no. CSB‐EL008770MO) in the serum and hippocampus tissue were measured by ELISA according to the manufacturer's protocol.

### 2.10 Statistical Analysis

The statistical analyses were performed using GraphPad Prism version 9.0. Data were first tested for normality. Three groups were analyzed and compared with each other using one‐way ANOVA with Dunn's correction followed by Tukey's post hoc comparison. Non‐parametric data were analyzed by Kruskal–Wallis test and Dunn's multiple comparison or Kolmogorov–Smirnov test for two‐group comparisons. The data were expressed as the mean ± SEM. *p *< 0.05 was considered to indicate a statistically significant difference.

## 3 Results

### 3.1 Irisin Improved Post‐Stroke Cognitive Performance

**FIGURE 1:**
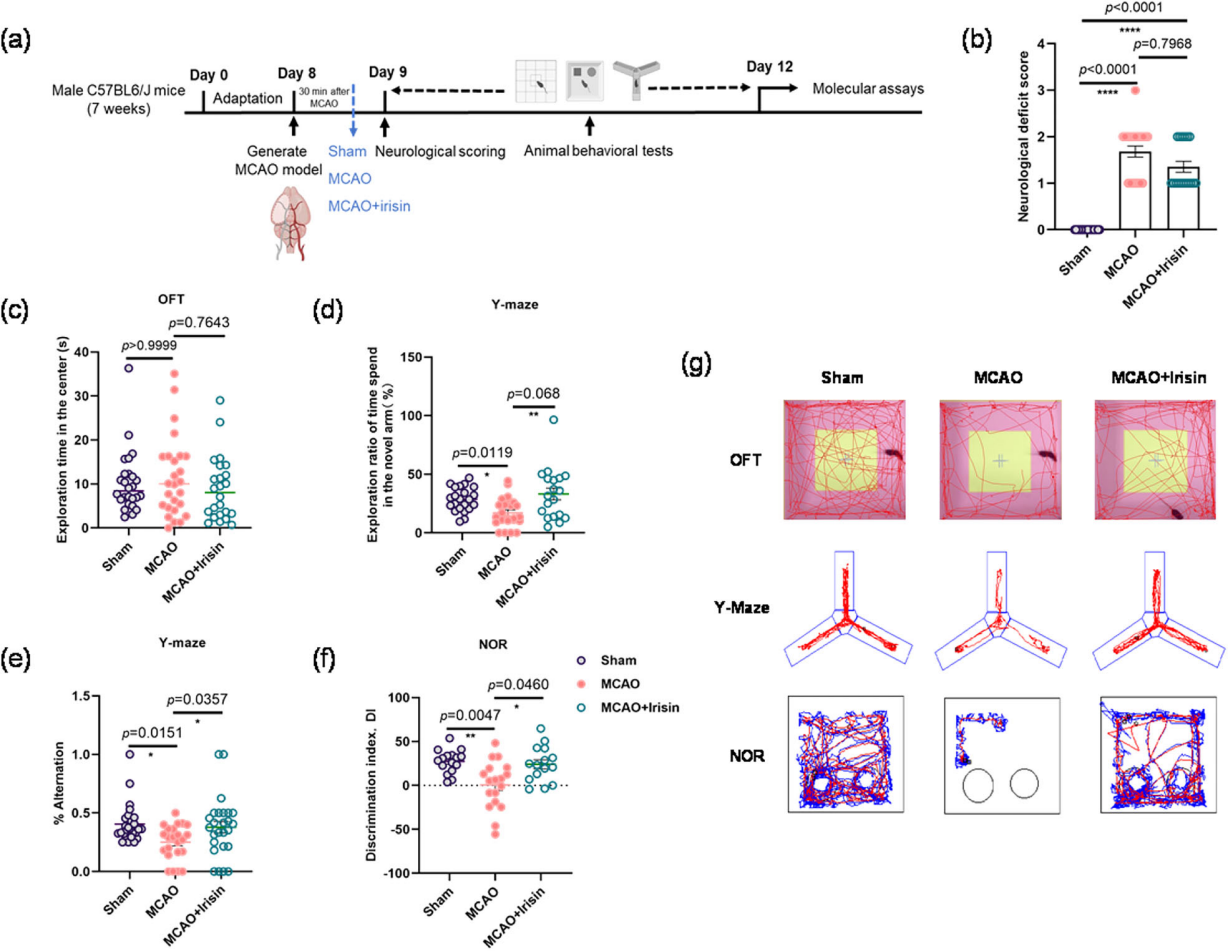
Effects of irisin on PSCI induced by the MCAO model mice. (a) Timelines of the experimental procedure. (b) Neurological deficit scores. One‐way analysis of variance followed by Kruskal–Wallis test with Dunn's correction showed significant differences (Kruskal–Wallis test statistic = 44.84, *p <* 0.0001) among the groups. *n* = 20, 22, and 17 mice in sham, MCAO, and MCAO + irisin groups, respectively. (c) Exploration time in the center in the open field over 5 min. One‐way analysis of variance followed by Kruskal–Wallis test with Dunn's correction revealed that there is the same locomotor activity (Kruskal–Wallis test statistic = 1.508, *p* = 0.4704) among the groups. *n* = 26, 26, and 24 mice in sham, MCAO, and MCAO + irisin groups, respectively. (d) Exploration of percentage (%) time spent in the novel arm. One‐way analysis of variance followed by Kruskal–Wallis test with Dunn's correction suggested decreased exploration (*p* = 0.0119) in MCAO mice, and irisin increased the exploration activity (*p* = 0.068) in irisin‐treated mice. *n* = 22, 24, and 19 mice in sham, MCAO, and MCAO + irisin groups, respectively. (e) Exploration of percentage (%) alteration in the novel arm. One‐way analysis of variance followed by Kruskal–Wallis test with Dunn's correction revealed that irisin plays a positive role (Kruskal–Wallis test statistic = 9.312, *p* = 0.0095) in MCAO mice. *n* = 27, 23, and 26 mice in sham, MCAO, and MCAO + irisin groups, respectively. (f) Discrimination ratios (or index) during the 5 min of exploration in the NOR tests. One‐way analysis of variance followed by Kruskal–Wallis test with Dunn's correction showed less interests (*p* = 0.0047) for the novel object in MCAO mice and irisin‐treated mice, showing more interest (*p* = 0.0460) for the novel object compared with MCAO mice. *n* = 16, 18, and 15 mice in sham, MCAO, and MCAO + irisin groups, respectively. (g) Representative track visualization in the OFT, Y‐maze, and NOR tests. The data are presented as the mean ± SEM. MCAO, middle cerebral artery occlusion; NOR, novel object recognition. *p < 0.05; **p < 0.01; ****p < 0.0001.

To determine the role of irisin in PSCI mice, we first evaluated the neurological scores on the PSCI model mice after 24‐h MCAO surgery to make sure the model is valid and reliable (Figure 1b). Both the PSCI mice and irisin‐treated PSCI mice showed a greatly higher neurological deficit score compared with that of the sham group (Figure 1b). There is no difference between MCAO injury and irisin‐treated mice (Figure 1b). However, all the groups have equal locomotor activity without any anxiety or depressive mood (Figure 1c,g).

Next, we determined whether irisin‐influenced MCAO‐induced learning and memory deficits. We used the Y‐maze and NOR tests to assess learning and memory performance in the mice. In the Y‐maze test, mice treated with irisin spent more time to explore in the novel arm (Figure 1d) and had a greater number of alternations in this area (Figure 1e). In the NOR test carried out after 24‐h surgery, mice who had received irisin treatment showed significantly increased spatial learning and memory (Figure 1f), whereas these abilities were reduced in the PSCI group (Figure 1f,g). These results suggest that irisin has a protective role against PSCI.

### 3.2 Irisin Reduces Infarct Volume and Inhibits the Number of Dead Neurons

**FIGURE 2:**
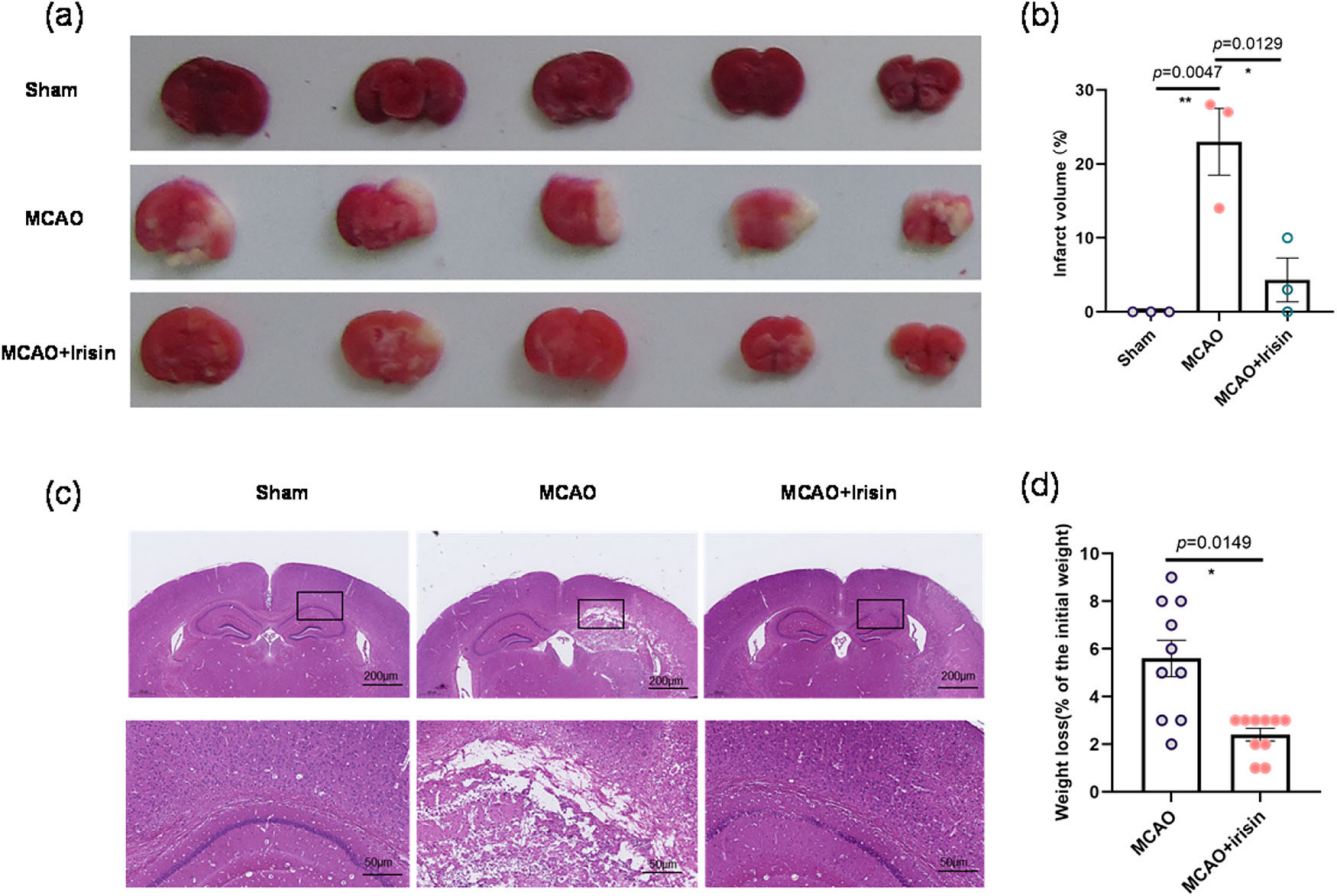
Histological and pathological changes associated with irisin treatment in MCAO model mice with cognitive impairment. (a) Representative images of TTC staining. (b) Quantification of the infarct volume in the different groups. One‐way analysis of variance followed by Tukey's post hoc test showed significant differences (*F* (2,6) = 15.39, *p* = 0.0043) among the groups. *n* = 3. (c) Representative images of HE staining. (d) Weight changes in the different groups. The *t*‐test followed by Kolmogorov–Smirnov test showed a great decreased wight loss (Kolmogorov–Smirnov *D* = 0.7000, *p* = 0.0149) in irisin‐treated mice. *n* = 10. Data are presented as the mean ± SEM. *p <  0.05; **p < 0.01.

TTC and HE staining were used in the study to determine whether treatment with irisin reduced pathological changes associated with PSCI in MCAO‐induced mice. The results showed clearly that the area of cerebral infarction was markedly increased in the MCAO injury group, but that the infarction volume was reduced in the mice treated with irisin (Figures 2a,b). Irisin also had a beneficial effect on the survival of hippocampus neurons by keeping the integrity of neuron cell or brain tissue structure (Figure 2c). Notably, irisin also decreased the body weight of the mice (Figure 2d), a result possibly consistent with their exercise characteristics. These results confirmed that irisin acted directly on PSCI.

### 3.3 Irisin Activates Endothelial Cells and Astrocytes Function in PSCI Model Mice

**FIGURE 3:**
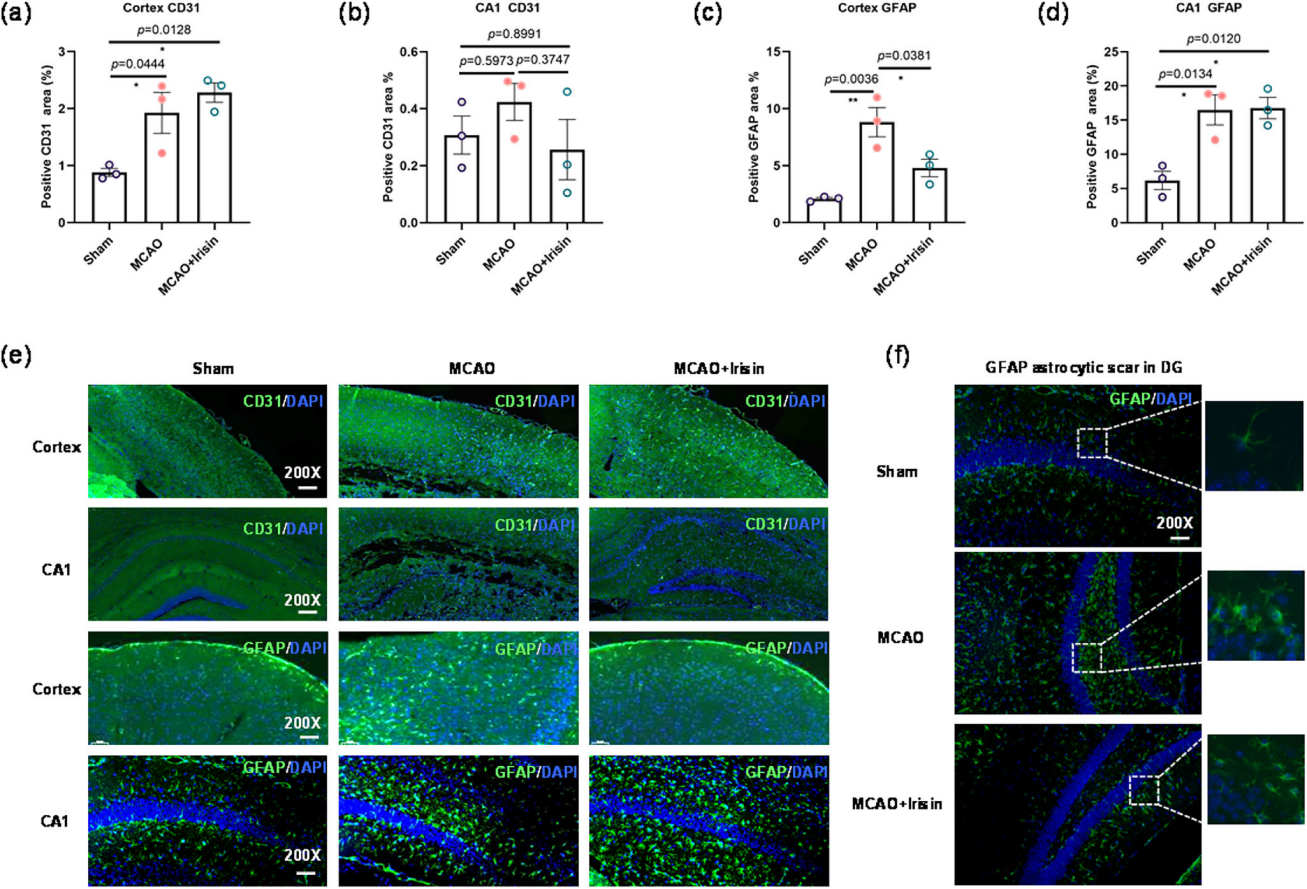
The effect of irisin treatment on astrocyte activity and angiogenesis in MCAO model mice. (a and b) Quantification of the CD31‐positive areas. One‐way analysis of variance followed by Tukey's post hoc test showed there is a significant difference (*F* (2,6) = 9.722, *p* = 0.0131) compared with the sham group in cortex CD31, and there is no difference (*F* (2,6) = 1.111, *p* = 0.3885) among the groups in the CA1 region CD31. (c and d) Quantification of the GFAP‐positive areas. One‐way analysis of variance followed by Tukey's post hoc test showed significant differences (*F* (2,6) = 15.38, *p* = 0.0044; *F* (2,6) = 12.07, *p* = 0.0079) among the groups. (e and f) Representative images of immunofluorescence staining for CD31‐ and GFAP‐positive cells. Scale bar, 200×. *n* = 3. Data are presented as the mean ± SEM. *p < 0.05; **p < 0.01.

Improved vascular remodeling and decreased inflammatory responses are two effective strategies for improving the outcomes of an ischemic stroke (Iadecola et al. 2019). We therefore evaluated the action of irisin on angiogenesis and neuroinflammation in the peri‐infarction area of the cortex and hippocampus in PSCI mice. Interestingly, mice treated with irisin had higher levels of the vascular endothelial cell marker CD31 in the cortex (Figure 3a) compared with those in the sham group (Figure 3a). However, no difference was observed in the CA1 area among the sham, the MCAO, and the irisin treatment groups (Figure 3b). Furthermore, MCAO caused astrocytic scar formation in the cortex around the infarct area and the dentate gyrus (DG) region in response to the injury (Figure 3e,f). However, irisin significantly decreased the production of GFAP in the cortex compared with the PSCI model group (Figure 3c) and increased it in the DG area as MCAO group (Figure 3d). Taken together, these results suggest that the production of CD31 and GFAP caused by irisin treatment was closely related to the activation of endothelial function and astrocyte‐neurons.

### 3.4 Irisin Improved Regulation of Endothelial Function After Post‐Stroke Cognitive Impairment via AMPK‐eNOS Signaling

**FIGURE 4:**
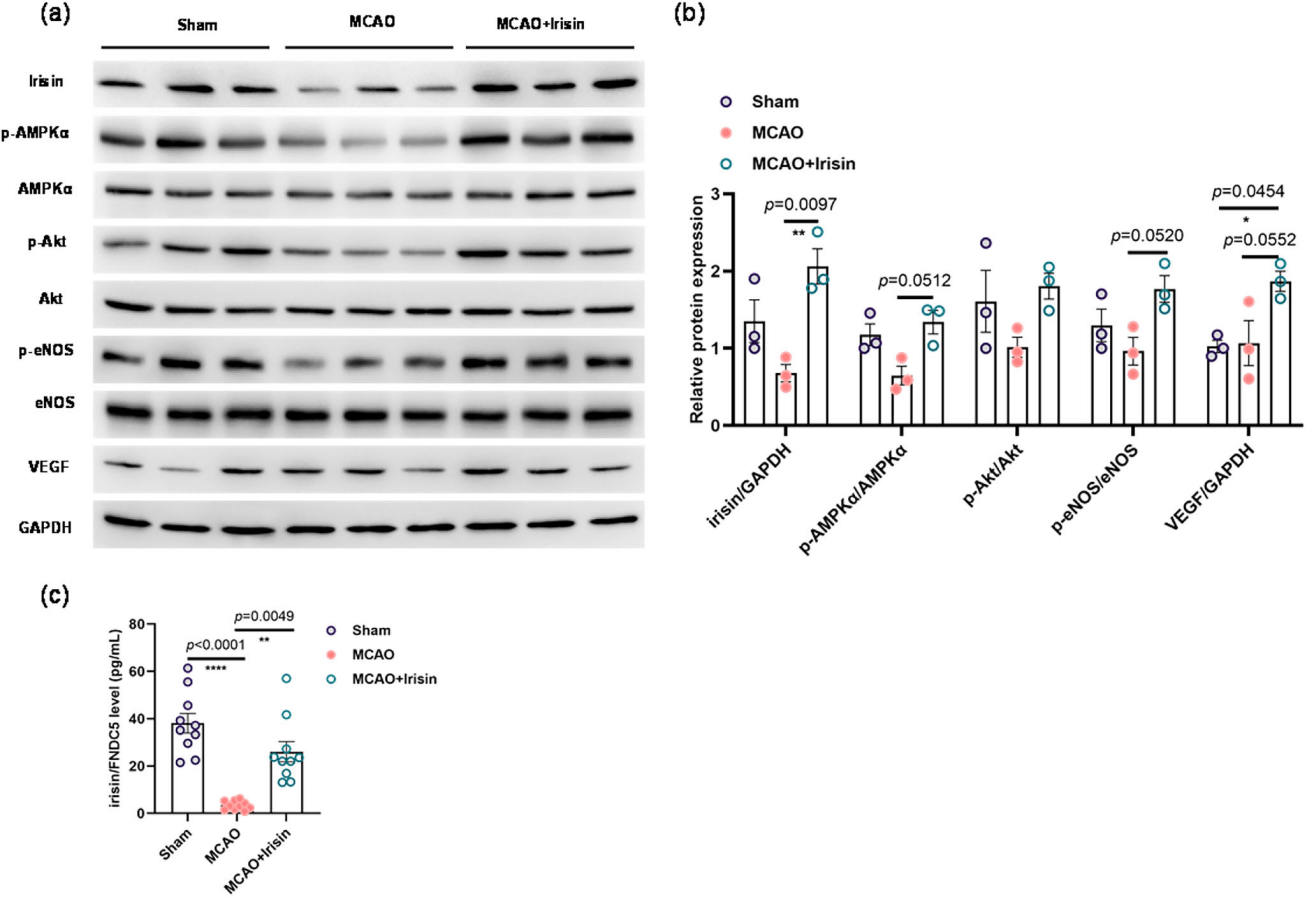
Irisin upregulated the AMPK‐eNOS signaling pathway in the hippocampus. (a) Representative images of the Western blotting analyses. (b) The expression levels of irisin, p‐AMPKα, p‐Akt, p‐eNOS, and VEGF. One‐way analysis of variance followed by Tukey's post hoc test and Kruskal–Wallis test with Dunn's correction showed that p‐Akt expression level has no difference (*F* (2,6) = 2.484, *p* = 0.1637) among the groups; p‐AMPKα, p‐eNOS, and VEGF were greatly decreased in MCAO mice compared with the sham group, and the expression level can be rescued by giving irisin treatment (*F* (2,6) = 10.13, *p* = 0.0119; Kruskal–Wallis test statistic = 5.956, *p* = 0.0250; *F* (2,6) = 4.652, *p* = 0.0603; *F* (2,6) = 6.289, *p* = 0.0337) among the groups. *n* = 3. (c) Concentration of irisin/FNDC5 in serum. One‐way analysis of variance followed by Kruskal–Wallis test with Dunn's correction showed that irisin expression was significantly decreased (*p <* 0.0001) in the MCAO mice, and irisin treatment showed the positive effects (*p =* 0.0049). *n* = 10 mice per group. Data are presented as the mean ± SEM. *p < 0.05; **p < 0.01; ****p < 0.0001.

To further understand the molecular mechanisms of irisin treatment in PSCI, we examined the expression levels of key markers for vascular endothelial function, VEGF, eNOS, AKT, and AMPKα (Figure 4a). Irisin appeared to promote the secretion of VEGF (Figure 4b). It can also promote the secretion of p‐AMPKα and p‐eNOS in the hippocampus with an approximate statistical difference (Figure 4b). Though there is no difference about the statistics for the AKT expression, the p‐AKT level seems to be downregulated in the MCAO injury group and tends to be restored in the irisin therapy group as the sham group (Figure 4b). It is worth noting that the serum irisin level was significantly decreased in PSCI mice compared with sham groups (Figure 4c), although the relative expression did not present the difference in WB assay (Figure 4b). These results suggest irisin has beneficial effects on PSCI, possibly due to activation of AMPK‐Akt‐eNOS‐VEGF signaling.

To confirm that the benefits of irisin on PSCI mice are mediated by the activation of eNOS and VEGF in an AMPK‐dependent manner, we treated different groups with an AMPK inhibitor (Compound C) and an eNOS inhibitor (L‐NAME). Compared to the irisin treatment group, the PSCI mice receiving the AMPK inhibitor showed significant declines in learning and memory, as demonstrated by the Y‐maze (Figure S1a–d) and the NOR test (Figure S1e,f). Additionally, levels of eNOS and VEGF proteins were reduced in the AMPK inhibitor group (Figure S2a). Interestingly, the eNOS inhibitor treatment did not result in significant changes in AMPK protein levels (Figure S2b). We also found a positive correlation between irisin expression levels and VEGF. Both the AMPK and eNOS inhibitors led to decreased VEGF expression in the hippocampus (Figure S2a,b), highlighting the interconnected roles of these pathways in cognitive function.

## 4 Discussion

This study investigated the therapeutic effects of irisin in a mouse model of PSCI. First, we showed that irisin rescued the impaired learning and memory ability in the Y‐maze and NOR tests 24 h after a stroke. Second, irisin contributed to a reduction in the size of the brain infarct area induced by ischemia and also ameliorated abnormalities in neuronal morphogenesis. Third, irisin activated cerebromicrovascular endothelial cells in the cortex and stimulated astrocytes in both the cortex and hippocampus regions in the brain. Finally, we showed that irisin significantly promoted the expression levels of VEGF in the hippocampus by stimulating the AMPK‐Akt‐eNOS signaling cascade.

Previous studies have suggested that approximately 25%–50% of patients with cognitive impairment are survivors of stroke and have a subtype of vascular lesions that are the second most common cause of dementia (Inoue et al. 2023; Iadecola et al. 2019). Therefore, we generated a mouse model of MCAO to imitate a cerebral ischemic event and then used the learning‐ and memory‐damaged animals as a PSCI model to measure neurological deficit scores and performance characteristics in the Y‐maze and NOR tests. Although there is still no effective pharmacological intervention for cognitive impairment patients, large evidence has found that physical exercise may delay the onset of AD (De La Rosa 2020), improve frailty and neuropsychological diseases (Izquierdo and Fiatarone Singh 2023), and enhance healthy human lifestyle. Therefore, the underlying physical exercise is growly being investigated recently. Irisin, one of the exerkines mainly from muscle and adipose tissue (Bao et al. 2022; Chow et al. 2022), plays a key role in neuroprotection (Maak et al. 2021). Here, we focus on the effects of irisin in PSCI mice and its potential molecular mechanisms.

Cerebral endothelial dysregulation contributes to dementia (Eisenmenger et al. 2023). It is known that cerebral endothelial cells and astrocytic end‐feet are two parts of the neurovascular unit (NVU) that influence brain homeostasis, the integrity of the blood–brain barrier (BBB), and CBF (Song et al. 2021; Yu et al. 2020). Therefore, in the current study, we measured the expression levels of CD31, a marker of microvascular density, and the area of positivity for glial fibrillary acidic protein (GFAP), a marker used to identify astrocytes in both the cortex and the hippocampus area (Figure 3e,f). Compared with the sham group, mice treated with irisin had an increased density of microvessels in the peri‐infarct cortex (Figure 3a) and angiogenesis in the hippocampus (Figure 4b). This suggested that irisin may restore brain functions by promoting neurovascular remodeling in the ipsilateral hemisphere. In addition, immune‐histochemical staining showed that the infarcted regions in the cortex and hippocampus were surrounded by high GFAP glial scar areas in the MCAO groups, but the peri‐infarct cortex revealed a decline in irisin group (Figure 3c). An excessive astrocyte reactivity was regarded as an abnormal progress on Aβ with early tau phosphorylation in preclinical AD (Bellaver et al. 2023). This means that irisin can promote the PSCI presentation by suppressing the formation of the GFAP glial scar. Interestingly, the expression of GFAP‐positive cells in the irisin treatment group was as much as in the PSCI model group (Figure 3d). Astrocytic phagocytosis has been confirmed to be necessary to cognitive function through maintaining proper hippocampal synaptic connectivity and plasticity (Lee et al. 2021). According to the astrocyte morphology (Figure 3f), the increased GFAP‐positive level in the irisin group in the hippocampus (Figure 3d) may be the activated astrocytes playing the neuroprotective role in PSCI. In fact, there are still uncertainties and controversies regarding the contribution of reactive astrocytes to CNS diseases, repair, and aging (Escartin et al. 2021). Therefore, for further study, combining with additional markers is needed (Escartin et al. 2021). As a whole, these findings suggest that irisin potentiates neurological recovery by regulating endothelial cell and astrogliosis.

There is evidence that irisin protects against BBB dysfunction, ameliorates endothelial dysfunction, and regulates blood pressure via the AMPK‐eNOS signaling pathway (Fu et al. 2016; P. Guo et al. 2019; Han et al. 2015). Hippocampal vascular dysfunction has a major role in hypertension and vascular cognitive impairment (Johnson 2023; Gannon et al. 2023). AMP‐activated protein kinase (AMPK) directly rescues endothelial dysfunction by activating AMPK‐eNOS (Sanz‐Gómez et al. 2022). We therefore evaluated whether irisin had beneficial effects in mice with PSCI by activating the AMPK‐Akt‐eNOS pathway. Our results showed that irisin tends to increase the expression levels of p‐AMPKα and p‐eNOS in the hippocampus, indicating that irisin may relate to the AMPK‐eNOS pathway. Notably, angiogenesis has been reported to be mediated via AMPKα1 activation induced by VEGF in an eNOS‐independent manner (Stahmann et al. 2010). Moreover, both Akt and eNOS are the downstream effectors that mediate VEGF‐initiated endothelial survival and angiogenesis (Sanz‐Gómez et al. 2022). We therefore also investigated whether irisin improved endothelial disorders in mice with PSCI by upregulating the expression levels of VEGF. Compared with the sham mice, irisin significantly increased the secretion of VEGF (Figure 4b). This suggested that irisin may be effective for protecting against post‐stroke neurovascular injury and underlines the mechanism related to the rescue of endothelial dysfunction caused by stimulation of the AMPK‐eNOS pathway.

In summary, this study demonstrated that irisin improves PSCI by reducing the volume of cerebral infarcts and promoting angiogenesis and maintaining the normal astrocytes and endothelial functions via the AMPK‐eNOS pathway.

## Author Contributions

**Hui–Hui Guo**: writing – original draft, software, formal analysis, investigation, data curation, validation, visualization, writing – review and editing. **Rui–Huan Pan**: validation, investigation, writing – review and editing, visualization. **Suk–Yu Yau**: writing – review and editing, supervision, conceptualization, project administration, methodology, visualization. **Mei–Feng Zheng**: investigation, formal analysis, writing – review and editing, validation, data curation. **Jun–Jie Liang**: writing – review and editing. **Ya–Xian Qiu**: writing – review and editing, validation, visualization, data curation. **Shan–Shan Jiang**: writing – review and editing, validation, data curation, formal analysis. **Xin–Yu Fu**: writing – review and editing, validation, data curation, formal analysis. **Hector Wing–Hong Tsang**: supervision, project administration, writing – review and editing, funding acquisition, resources. **Hai–Ning Ou**: resources, funding acquisition, writing – review and editing, project administration, supervision, conceptualization, methodology.

## Ethics Statement

The animal study protocol was approved by the Ethics Committee of the Hong Kong Polytechnic University (PolyU) Shenzhen Research Institute Committee on Animal Care (ASESC Case No.: 20–21/303‐RS‐R‐NSFC).

## Conflicts of Interest

The authors declare no conflicts of interest.

## Peer Review

The peer review history for this article is available at https://publons.com/publon/10.1002/brb3.70767.

## Supporting information



brb370767‐sup‐0001‐SuppMat.pdf: Figure S1

## Data Availability

The data that support the findings of this study are available from the corresponding author upon reasonable request.
