# Supplementary material for: Irisin/FNDC5 Regulates Endothelial Function to Improve Post‐Stroke‐Induced Cognitive Dysfunction by Stimulating AMPK‐eNOS Signaling
Source: Brain Behav. 2025 Sep 15;15(9):e70767. doi: 10.1002/brb3.70767 (PMC12434608; doi:10.1002/brb3.70767)
Supplement: Supplementary file 1 — brb370767‐sup‐0001‐SuppMat.pdf: Figure S1 [file BRB3-15-e70767-s001.pdf]

Supporting Information

Supplemental results

Irisin improved regulation of endothelial function after post-stroke cognitive impairment via AMPK-eNOS signaling

Figure S1.

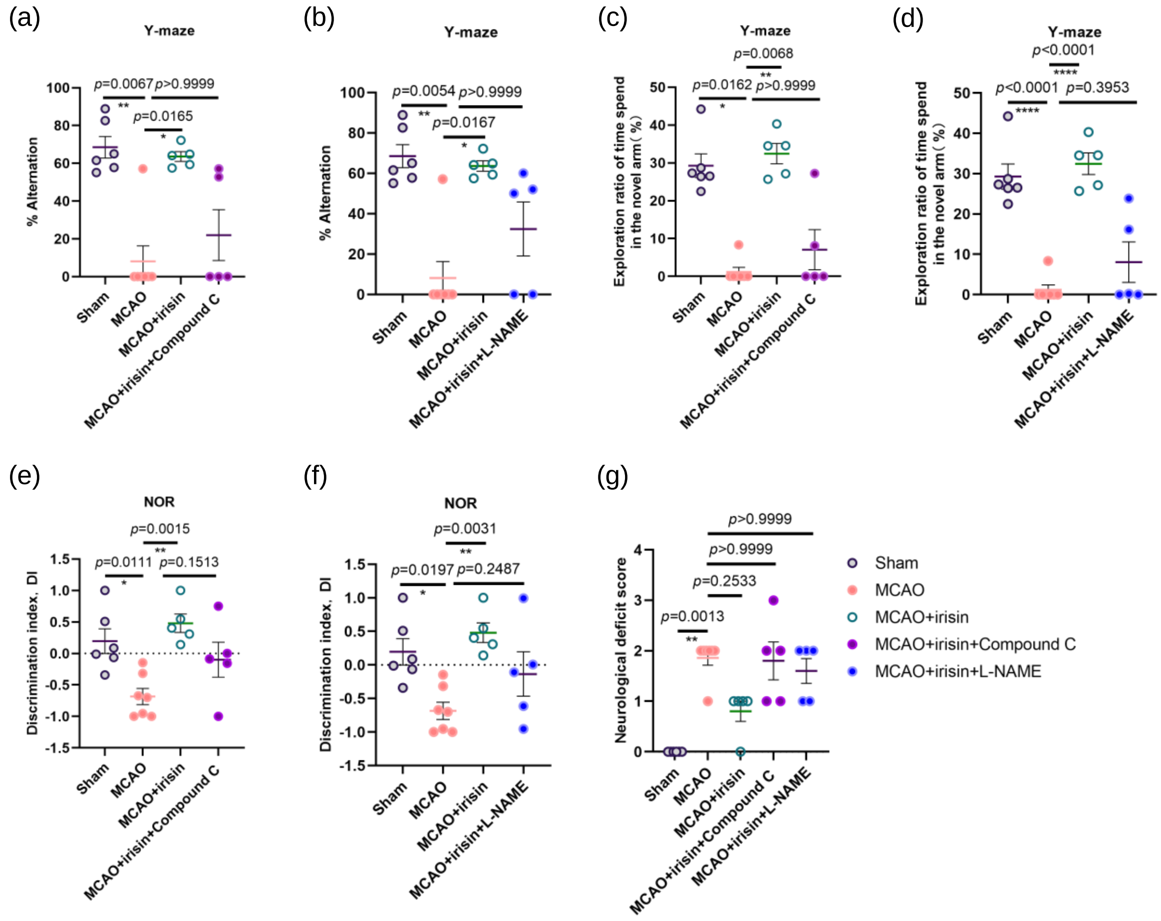

Figure S1. AMPK inhibitor decreased effects of irisin on PSCI by AMPK-eNOS signaling. (a and b) Exploration of percentage (%) alteration in the novel arm. One-way analysis of variance followed by Krus-kal-Wallis test with Dunn's correction showed that both compound C (*P* >0.9999 vs. MCAO) and L-NAME treatment mice (*P* >0.9999 vs. MCAO) inhibit irisin positive effects. (c and d) Exploration of percentage (%) time spent in the novel arm. One-way analysis of variance followed by Krus-kal-Wallis test with Dunn's correction revealed that both compound C (*P* >0.9999 vs. MCAO) and L-NAME treatment mice (*P* =0.3953 vs. MCAO) decreased the irisin treatment effects. (e and f) Discrimination ratios (or index) during the five minutes of exploration in the NOR tests. One-way analysis of variance followed by Tukey's post hoc test showed that irisin treatment effects lost in the MCAO+irisin+Compound C group (F (3,19) = 7.582, *P* =0.1513 vs. MCAO+irisin) and in the MCAO+irisin+L-NAME group (F (3,19) = 6.507,

P =0.0033). (g) Neurological deficit scores. One-way analysis of variance followed by Kruskal-Wallis test with Dunn's correction showed that PSCI model can be used in this study (Kruskal–Wallis test statistic = 19.48, P =0.0013 vs. Sham). n = 6, 7, 5, 5 and 5 mice in the Sham, MCAO, MCAO + inrisin, MCAO + inrisin + Compound C and MCAO + inrisin + L-NAME respectively. *P< 0.05; **P<0.01 and ****P<0.0001. The data are presented as the mean ± SEM. MCAO, middle cerebral artery occlusion; NOR, novel object recognition; Compound C, AMPK inhibitor; L-NAME, eNOS inhibitor.

Figure FS2

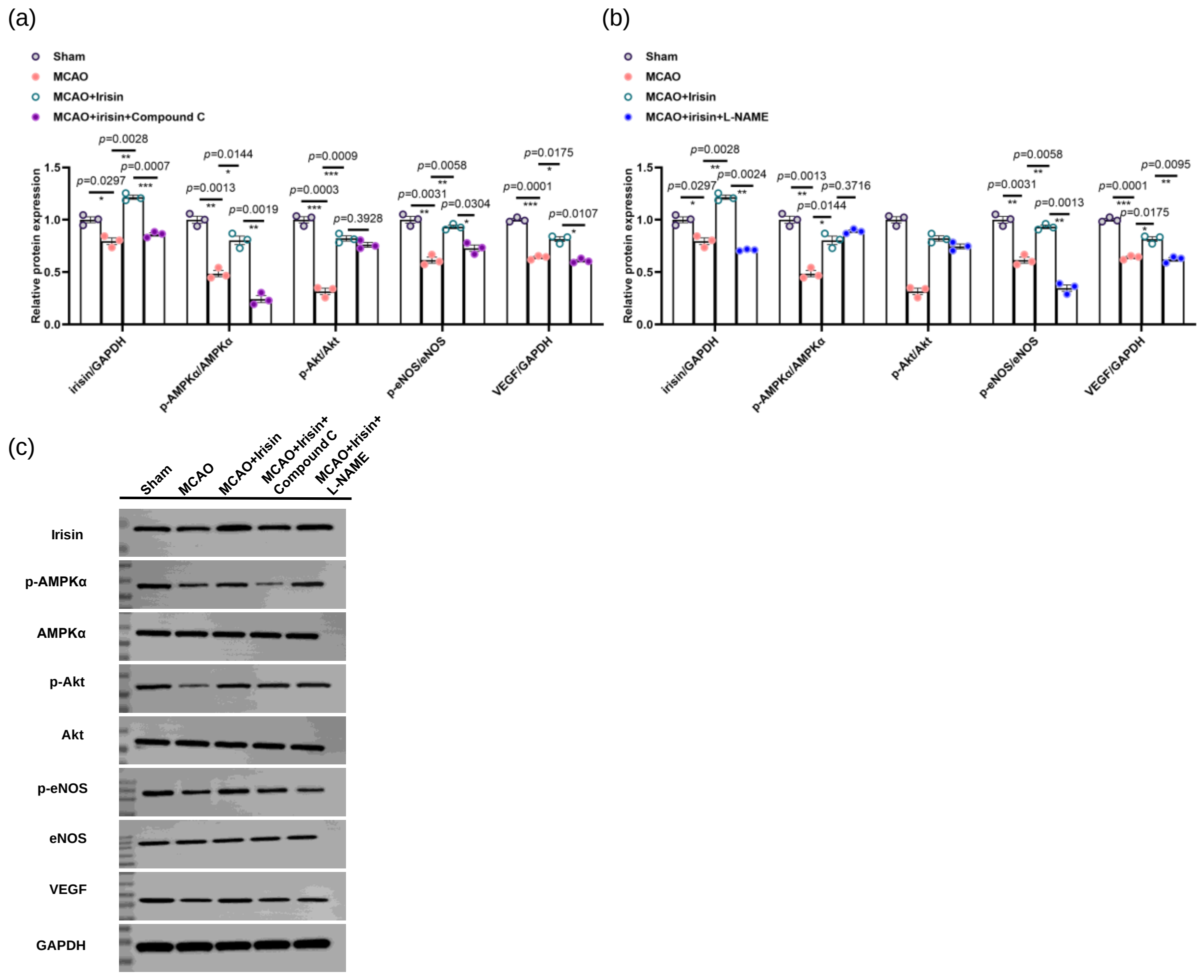

Figure FS2. Irisin benefits on PSCI dependent on AMPK-eNOS signaling. (a) The expression levels of irisin, p-AMPKα, p-Akt, p-eNOS and VEGF in PSCI with AMPK inhibitor or eNOS inhibitor treatment mice. Two-way analysis of variance followed by Tukey's multiple comparisons test: AMPK inhibitor: P (different group) < 0.0001, F (3,8) = 249.7; P (same mark) < 0.0001, F (2.719,21.75) = 92.39; P (*interaction*) < 0.0001, F (12,32)

= 29.29; eNOS inhibitor: P (different group) < 0.0001, F (3,8) = 251.7; P (same mark) $<$ 0.0001, F (2.625,21.00) = 47.83; P (*interaction*) < 0.0001, F (12,32) = 39.99. (b) Representative images of the Western blotting analyses. n = 3 mice per group. Data are presented as the mean ± SEM. *$P<0.05$, **$P<0.01$ and ***$P<0.001$.
